# Diet in Diabetes

**Published:** 1900-05-26

**Authors:** 


### DIET IN DIABETES.

In arranging the diet for a diabetic one must consider the patient as well as the disease, and must provide food enough to maintain his nutrition and to supply the waste caused by his disease. Moreover, it is important not to do this by giving an excessive quantity of albuminous food, the giving of which in such cases has no doubt been the cause of the untoward results which are alleged to have followed the use of strict diabetic diet in some cases. Rubner has shown that a man doing slight work consumes for each kilogramme of body-weight an amount of food equivalent to 34·9 heat units. Putting this into English form, Dr. Saundby says that a person weighing 10 stone on light work requires daily an alimentary value of 2,450 heat units. In addition to this, if he is to keep his weight a diabetic subject must receive food enough to supply the sugar which he excretes unconsumed, which may be roughly calculated on the basis of each 15 grains of sugar excreted being equivalent to 4 heat units expressed in food. To save the necessity of working out the heat units and their equivalents in foods of various kinds for each case, Dr. Saundby has arranged a series of three diets containing progressive quantities of carbohydrate ingredients, which, we think, will be found very useful in practice. Whichever diet is being used one knows fairly exactly how many grains of carbohydrate are being taken, and by adding to that the amount formed by the metabolism within the body (which equals twice the weight of the urea which is excreted), one has the total of the carbohydrate supplied to the tissues, and by subtracting from this the total amount of the sugar excreted it is easy to see how much of the carbohydrate is being appropriated by the tissues. This is a matter of considerable importance, because in many cases it may be worth while to be more liberal with the carbohydrates rather than increasing the proteid elements in the diet, even though the glycosuria may be increased, if at the same time the amount of carbohydrate assimilated is also largely increased; while if it should be found that all such increase in the carbohydrates taken runs off by the kidneys in the form of sugar, there clearly would be no compensating advantage to repay the risks, and the strict diet must be continued. The diets are as follows:

Diet A.—This is worked out for a patient requiring 2,400 heat units—about what would be required by a man weighing nearly 10 stone, and doing slight work—and is so arranged as to be as far as possible free from carbohydrates, the total amount contained in the green vegetables and bread-substitutes not exceeding 500 grains of sugar-forming material. It consists of six ounces of cooked meat; two ounces of fat bacon; two eggs; two ounces of cream; three ounces of butter; three ounces of cheese; six ounces of green vegetables; nine ounces of Callard's brown loaf, biscuits, and sponge cakes; half a pint of bouillon; one pint of tea; half a pint of claret; two ounces of Scotch whisky. Out of the above articles it is possible to arrange breakfast, luncheon, tea, and dinner; and it is an advantage to make the three principal meals nearly equal, so as not to take too much at any one time.

Diet B.—This gives 2,569 heat units at the cost of giving 1,325 grains of carbohydrates. It consists of two ounces of bacon; three eggs; two ounces of butter; two ounces of cheese; one pint of milk; six ounces of cooked meat; six ounces of Callard's bread, &c.; six ounces of green vegetables; six ounces of potatoes; one pint of tea; half a pint of bouillon; half a pint of claret; two ounces of whisky.

Diet C.—Gives 3,011 heat units at the cost of giving 2,466 grains of carbohydrates. It consists of two ounces of fat bacon; three eggs; two ounces of butter; three ounces of cheese; one ounce of cream; one pint of milk; six ounces of cooked meat; six ounces of green vegetables; six ounces of potatoes; four ounces of toast; two ounces of Callard's sponge cake, four ounces of fruit; half a pint of bouillon; one pint of tea; four ounces of coffee; half a pint of claret; two ounces of whisky.

